# Neighbourhood Factors and Depression among Adolescents in Four Caribbean Countries

**DOI:** 10.1371/journal.pone.0095538

**Published:** 2014-04-23

**Authors:** Gillian A. Lowe, Garth Lipps, Roger C. Gibson, Sharon Halliday, Amrie Morris, Nelson Clarke, Rosemarie N. Wilson

**Affiliations:** 1 Department of Community Health and Psychiatry, The University of the West Indies, Mona, Kingston, Jamaica; 2 Department of Sociology, Psychology and Social Work, The University of the West Indies, Mona, Kingston, Jamaica; 3 Ministry of Health, Social Services, Community Development, Culture & Gender Affairs, Government of St. Kitts and Nevis, Basseterre, St. Kitts and Nevis; 4 Trinity School of Medicine, Kingstown, St. Vincent and the Grenadines; 5 School of Clinical Medicine and Research, The University of the West Indies – Bahamas, Nassau, The Bahamas; Wayne State University, United States of America

## Abstract

**Background:**

Past research suggests that perceived neighbourhood conditions may influence adolescents' emotional health. Relatively little research has been conducted examining the association of perceived neighbourhood conditions with depressive symptoms among Caribbean adolescents. This project examines the association of perceived neighbourhood conditions with levels of depressive symptoms among adolescents in Jamaica, the Bahamas, St. Kitts and Nevis, and St. Vincent.

**Methods:**

Adolescents attending grade ten of the academic year 2006/2007 in Jamaica, the Bahamas, St. Vincent, and St. Kitts and Nevis were administered the Neighbourhood Characteristics Questionnaire along with the BDI-II. Social cohesion, attachment to the neighbourhood, neighbourhood quality, neighbourhood crime, and neighbourhood disorder scales were created by summing the relevant subscales of the Neighbourhood Characteristics Questionnaire. Multiple regression analyses were used to examine the relationships of perceived neighbourhood conditions to depressive symptoms.

**Results:**

A wide cross-section of tenth grade students in each nation was sampled (n = 1955; 278 from Jamaica, 217 from the Bahamas, 737 St. Kitts and Nevis, 716 from St. Vincent; 52.1% females, 45.6% males and 2.3% no gender reported; 12 to 19 years, mean = 15.3 yrs, sd = .95 yr). Nearly half (52.1%) of all adolescents reported mild to severe symptoms of depression with 29.1% reporting moderate to severe symptoms of depression. Overall, Jamaican adolescents perceived their neighbourhoods in a more positive manner than those in the Bahamas, St. Vincent and St. Kitts and Nevis. Results of a series of hierarchical multiple regression analyses suggested that a different pattern of neighbourhood factors for each island were associated with depressive symptoms. However, neighbourhood factors were more highly associated with depressive symptoms for Jamaican students than for students in the other three islands.

**Conclusions:**

Neighbourhood factors appear to be partially associated with adolescents' self-reports of depressive symptoms. However, other factors may mitigate this relationship.

## Introduction

A Nigerian proverb states “it takes a village to raise a child” [1]. This saying embodies the essence of findings from social research which suggests that the social networks, norms and resources of a neighbourhood may have a significant impact on human development and behaviour [2], [3]. The proverb also has resonance with health issues, the implication being that in order for a child to transition into a healthy and well-adjusted adult, the entire community must play a part. Research exploring the association between neighbourhood characteristics and mental health has shown significant associations between the social and physical characteristics of a community on the one hand and mental health outcomes in both adults and children on the other [4]–[7]. Duncan and Raudenbush [8] posit that the impact of the neighbourhood may be particularly relevant for adolescents as generally they tend to make a transition from spending more time at home to spending more time away from home and interacting with neighbourhood-based resources. Despite this literature, little research has examined the role that neighbourhoods may have on Caribbean adolescents' mental health. This research project explores the association of five neighbourhood factors with depressive symptoms for adolescents living in four Caribbean nations. Results of this research may increase understanding of the factors which may influence the development of depressive symptoms in adolescents, as well as inform strategies to deal with this issue.

### Research and theory regarding the influence of neighbourhoods

Neighbourhoods are complex ecological settings whose characteristics and interactions are not easily understood, predicted or measured. Research in this area has tended to be guided by predetermined parameters informed by social theory. This includes the pivotal social disorganization theory of Shaw and McKay [9]. Sampson and Groves [10] tested the real-life applicability of this theory and confirmed that social disorganization, as exemplified by the inability of a community to support the maintenance of common values and social control, resulted in negative outcomes. More specifically, inadequate social networks, poorly supervised adolescents, and the absence of strong community organizations predicted high rates of crime and delinquency. Jencks and Mayer [2] also cited the importance of neighbourhood organizations and networks for individual outcomes. Other central aspects of their theoretical framework regarding the linkage between neighbourhoods and individual outcomes are the influence of role models, peer beliefs, competition for scarce resources and social inequalities. Bronfrenbrenner [11] proposed additional salient concepts which have guided some neighbourhood research. These include the dynamic interaction of various environments (e.g. home, neighbourhood, school) and how changes over time in all of these areas, and in their inter-relationships, may have an effect at the level of the individual.

As in other regions of the world, Caribbean countries have communities with diverse characteristics. Notwithstanding, there may be some general features of Caribbean societies that are relevant across most neighbourhoods. For example, Jamaica has been noted to have a strong, inclusive culture which rapidly assimilates peoples from other ethnicities such as African, Indian, Chinese, Middle Eastern and European backgrounds into a new, dominant Jamaican culture [12]. Additionally, there is an overarching, highly stratified social and educational system in Jamaica whereby people tend to become segregated by socio-economic status and social class, but less so by ethnicity. The inclusive culture may lend itself to a high propensity for social networks as described by Sampson and Groves [10] at the neighbourhood level. However, the social stratification may foster inequalities and competition for scarce resources as described by Jencks and Mayer [2]. In many ways, the cultures of St. Vincent and St. Kitts and Nevis are similar to that of Jamaica, but their neighbourhoods are less highly stratified by social class [13], [14]. In the Bahamas, similar social characteristics apply, but their highly structured school districts may tend to more concretely define and delimit neighbourhoods since this system of going to school where you live restricts the coming together of youth from diverse geographical communities.

### The relationship of neighbourhoods to mental health

Neighbourhood characteristics in previous research have been associated with specific mental issues such as depression [5], [15], [16]. Gary et al [5] showed that the perception of severe community problems was significantly associated with depression among adults in a racially diverse community in the United States. Similar findings have also been found in children and adolescents as exemplified by the work of Butler et al [15] who found that at the neighbourhood level, both poor physical infrastructure and poor social support were linked to depression in this age-group (6–17 years old). Aneshensel and Sucoff [4] have also shown an association of both lower socioeconomic status as well as perceptions of one's neighbourhood as being threatening with adolescent depression. Thus, both objective (e.g. socioeconomic status, physical infrastructure) and subjective (e.g. perceptions of threats in the environment) characteristics of neighbourhoods may be linked to depression. It is important to take both types of measures into consideration in any attempt to obtain a holistic understanding of what the neighbourhood means to the individual and how this may impact his/her mental health. Unfortunately, adolescents' perceptions of their neighbourhoods and how these perceptions may influence their mental health have not been well studied in the Caribbean.

### Extension of past research to the Caribbean context

Past research among adolescents in the Caribbean has shown a high prevalence (24.5% to 40.6%) of moderate to severe depressive symptoms [17]–[20]. The high rate of depressive symptoms in Caribbean adolescents is likely to be associated with a high burden of disease resulting from both short-term and long-term effects of being depressed. Recognized short-term effects of adolescent depression include a heightened risk of substance abuse, attempted suicide, unplanned pregnancies and academic underachievement [21]. Unfortunately, the onset of mental disorders in adolescence is also recognized as a harbinger of mental health problems in adulthood [22].

Given the far-reaching implications of adolescent depression, strategies aimed at curtailing the problem are desirable. Such strategies may be informed by an understanding of various factors that are associated with the condition. Little is known about the role of neighbourhoods in the development of depressive symptoms in Caribbean adolescents and the unique social and cultural characteristics of the Caribbean make the extrapolation of research findings from other settings questionable. As such, using Caribbean adolescents' own perceptions of their neighbourhoods, we sought to explore the association of social cohesion, level of attachment, neighbourhood quality, crime and neighbourhood disorder with levels of depressive symptoms. We hypothesized that greater levels of depressive symptoms would be found among youth who perceived their communities as being of low quality, having high levels of crime and disorder and low levels of social cohesion and attachment. However, because Caribbean countries have cultures which are similar in many respects, but also different in others, we also hypothesized that these neighbourhood factors would be moderated by the Caribbean country that adolescents live in.

## Materials and Methods

### Sample

Grade ten students from four Caribbean islands - Jamaica, St. Vincent, St. Kitts and Nevis and the Bahamas - were chosen to take part in the research project. Our obtained sample roughly approximated the gender distribution of the official school population reported by UNESCO [23]. These countries are English speaking independent nations which are part of the British Commonwealth. They are governed by constitutional monarchies with democratically elected legislatures. Jamaica gained its independence in 1962, while St. Vincent and the Bahamas gained their independence in the 1970's and St. Kitts gained its independence in 1983.

Jamaica is 10,991 square kilometres in size, with a population of approximately 2,700,000 people. The majority of the Jamaican population are of African descent (91%), while significantly less (6%) are of mixed race and the remainder are of unknown or other ethnicities [24].

St. Vincent and the Grenadines is 389 square kilometres in size and has a population of approximately 103,000 people. The majority of the population are of African (66%) or mixed descent (19%), with the remainder either East Asian (6%), European (4%), Carib-Amerindian (2%) or some other ethnicity (3%) [25].

St Kitts and Nevis is 261 square kilometres in size with a population of approximately 50,000 people who are predominately of African descent (90.4%) with a small number of the population who are mixed race (5%), Indo-Pakistani (3%), or European (1% British, Portuguese, or Lebanese descent) [25], [26].

The Bahamas, a multi-island archipelago, is 13,880 square kilometres in size. It has a population of approximately 347,000 people, with 85% of the population of African descent, 12% European and 3% Asian and Hispanic descent [27].

In each country we asked the local researchers to identify two types of schools based on guidance from their Ministry of Education – socially elite high schools and non-elite high schools. This purposive strategy of sampling for heterogeneous instances [28] was utilized to highlight the differences in academic and social prestige of schools and students' emotional health and perceptions so as to maximize the effect sizes of the data analyses.

Prior to the date of data collection, the local researcher liaised with the school principal for each school in each country to obtain their consent for their school to take part in the research. Prior to this meeting consent forms were given to students for their parents to complete and return on the day of data collection. The schools distributed and collected the parental consent forms, and on the day of data collection allowed the researchers' access to students in the classrooms. All students present on the day of data collection had signed parental consent forms.

Participants expressed a strong interest in the study and were highly motivated to take part in the research. As such, no participants refused to take part in the study. However, the study was school-based and those adolescents who were not present on the day of data collection were not included in the sample of each country. The demographic features of students by gender, maternal education, and age for each country can be seen in Table 1.

**Table 1:** Demographic features of sampled adolescents.

		Jamaica	Bahamas	St. Kitts and Nevis	St. Vincent	Chi-Square Test
		N (%)	N (%)	N (%)	N (%)	54.19*
Gender	Male	115 (41.4)	90 (41.5)	352 (47.8)	332 (46.4)	
	Female	145 (52.2)	114 (52.5)	371 (50.3)	384 (53.6)	
	Missing	18 (6.5)	13 (6.0)	14 (1.9)	0 (0.0)	
Maternal Education	Secondary or Less	130 (46.8)	79 (36.4)	437 (59.3)	487 (68.0)	135.04*
	Post-Secondary	148 (53.2)	138 (63.6)	204 (27.7)	177 (24.7)	
	Missing	0 (0.0)	0 (0.0)	96 (13.0)	52 (7.3)	
Age	13	0 (0.0)	44 (20.3)	0 (0.0)	0 (0.0)	689.60*
	14	40 (14.4)	89 (41.0)	72 (9.8)	66 (9.2)	
	15	179 (64.4)	64 (29.5)	309 (41.9)	342 (47.8)	
	16	59 (21.2)	20 (9.2)	279 (37.9)	191 (26.7)	
	17	0 (0.0)	0 (0.0)	77 (10.4)	117 (16.3)	

* p<.001

Students from the lower grades were not selected for this study as past research has indicated they were more likely to experience emotional problems due to issues of educational and developmental transitions [29]. Students from the eleventh and twelfth grades were not selected for the project because they were engaged in preparation for the Caribbean Secondary Education Council Examinations which are externally based, high stakes achievement tests [30]. The results of those tests determine students' future academic and career paths [30]. Consequently, students in those grades were expected to be severely stressed [31].

#### Jamaica

Jamaican high schools are stratified into socially elite, traditional and socially non-elite, non-traditional schools. This categorization parallels Jamaica's socio-economic stratification [32]. Students are assigned to either traditional or non-traditional high schools based on their exit examination performance at the end of elementary school. A cross-section of grade ten students from traditional and non-traditional high schools in urban and rural Jamaica were sampled (n = 278 students; 41% males, 52% females and 7% not stated; age 14 to 16 years, mean = 15.0 yrs+0.6 yr: Table 1). The schools sampled included a traditional urban high school, a non-traditional urban high school, a rural traditional high school and a non-traditional rural high school.

Traditional high schools are long standing (greater than 50 years of age), religious based institutions which originally were founded to educate the children of the middle class [33]. Non-traditional high schools were established more recently by the Government of Jamaica, many of them in the post-independence era (less than 50 years ago). They do not have a strong religious background and the curriculum is not as rigorous as that in the traditional high schools.

#### St. Vincent

Data were collected from 716 grade ten students attending eight secondary schools across the island of St. Vincent (Table 1). A local researcher (APM) selected the schools based on discussions with officials of the Ministry of Education of St. Vincent. The school system in St. Vincent parallels that of Jamaica in many features, including the presence of socially elite, traditional and socially non-elite, non-traditional high schools. The Ministry suggested non-traditional and traditional high schools to be sampled. Schools were selected such that three traditional and five non-traditional schools were sampled. Of the eight schools sampled, two were boys' schools, two were girls' schools and the remaining four were co-educational schools. Students within each school were randomly sampled to take part in the study. Of the 716 participants sampled, 384 (54%) were female and 332 (46%) were male (Table 1). Students ranged in age between 14 and 18 years (mean = 15.5 years, sd = 1.0).

#### St. Kitts and Nevis

The researchers contacted officials within the Ministry of Education regarding the selection of schools. Because of the high level of interest in the findings of the study, the Ministry of Education requested that all schools in St. Kitts and Nevis be sampled. As such, a near census of grade ten students attending all high schools in Saint Christopher (St. Kitts) and Nevis were surveyed (n = 737; Table 1). A list of all schools providing secondary education in St. Kitts and Nevis was obtained from the Ministry of Education. Schools were visited and all grade ten students in attendance on that day were surveyed. The sample consisted of nearly equal percentages of female (50%) and male (48%) students with 2% of the sample not reporting their gender. Students in our sample ranged from 13 to 17 years of age (mean = 15.5 yrs+0.8 yr: Table 1).

#### The Bahamas

A sample of 217 adolescents attending the tenth grade were sampled from four high schools on New Providence Island (42% males, 52% females and 6% not stated; age 13 to 16 years, mean = 14.3 yrs+0.9 yr: Table 1). The schools were sampled such that they included elite and non-elite schools present on New Providence Island.

### Measures

#### Beck Depression Inventory - II (BDI-II)

The Beck Depression Inventory (BDI-II) [34] is a 21 item questionnaire which examines the cognitive, behavioural, affective and somatic symptoms of depression. Each item of the BDI-II is comprised of a series of rank ordered statements measuring a symptom of depression. Associated with each statement are a series of response options, which are assigned a score from 0 to 3, reflecting the severity of the symptoms. Students were asked to circle the number associated with the response option that most accurately describes their feelings during the past two weeks. Previous research suggests that the BDI-II is reliable in North American samples of adults [34]. Studies using both non-clinical [34], [35] and clinical samples of adolescents [36] have reported acceptable internal consistency reliabilities, with coefficient alphas ranging from .87 to .94. Within a sample of Jamaican adolescents the BDI-II had a high internal consistency reliability (alpha = .87; [37], [38]). In the current sample the BDI-II was found to have a high internal consistency reliability (alpha = .88). Past research suggests that the BDI-II is valid across different cultures [35], [39], even in cultures that place a high stigma on psychological problems [40]. Based on their BDI-II scores, adolescents in this study were divided into minimal (13 or less), mild (14 to 19), moderate (20 to 28) or severe (29 or higher) symptoms of depression. While depression is a clinical diagnosis requiring professional assessment of adolescents by a clinical psychologist or psychiatrist, the BDI-II simply provides information on levels of depressive symptoms. Some youth, but not all, who have high BDI-II scores may be judged by clinicians to have major depressive disorder.

#### Neighbourhood factors

Assessment of adolescents' perceptions of their local community was collected using a modified version of the Neighbourhood Characteristics Questionnaire (NCQ; [41]). Changes to the questionnaire involved modifying the perspective from that of a parent to an adolescent. For example, the question “There are adults in the neighbourhood that children can look up to” was changed to “There are adults in the neighbourhood that I can look up to”.

The 38 items which made up the NCQ formed five sub-scales: Social cohesion, attachment to the neighbourhood, neighbourhood quality, neighbourhood crime, and neighbourhood disorder. The social cohesion subscale measured the extent to which people in the neighbourhood assisted and socialized with each other. For example, participants were asked if neighbours do favours for each other. All items were responded to using a dichotomous yes or no format with total scores having a possible range of 0 to 7. In this project, this subscale had an acceptable internal consistency (alpha = 0.67). Total scores on this subscale were calculated by summing scores for all of the items.

The attachment to the neighbourhood subscale examined the extent to which participants felt a part of their neighbourhood and their intentions of remaining in the neighbourhood. For example, participants were asked how long their family intended to stay in the neighbourhood and if they felt at home on their side of their street. All items making up this sub-scale were responded to using a combination of 1 to 5 Likert type response options, or dichotomous yes/no response options. The scores on the attachment to neighbourhood subscale had a possible range of 5 to 28. The attachment to neighbourhood sub-scale had an acceptable internal consistency (alpha = 0.64). As with the previous subscale, the total scores on this subscale were calculated by summing scores for all of the items.

The neighbourhood quality subscale assessed the quality of life available in the neighbourhood and the extent to which the neighbourhood is in decline. For example, participants were asked to rate the quality of their neighbourhood as a place to live and the extent to which it had become worse in the past year. The four items which made up this subscale were all responded to using a 1 to 5 Likert type format. The scores for this subscale had a possible range of 4 to 20. The neighbourhood quality sub-scale had an internal consistency reliability of 0.68.Total scores on this subscale were calculated by summing scores for all of the items. High scores on the subscale represent poorer perceived neighbourhood quality.

The nine items comprising the neighbourhood crime subscale examined respondents' perceptions of the safety of their neighbourhood. Respondents were asked whether a number of issues were a problem in their neighbourhood. Included in the list of problems were fights with a weapon, youth gang conflict, burglary of homes, and assault by strangers. All items were responded to using a yes/no format, with the total scores on this subscale ranging from 0 to 9. The internal consistency of this subscale was 0.72. Total scores on this subscale were calculated by summing scores for all of the items.

The neighbourhood disorder subscale assessed the extent of organization in the community. Items which made up this sub-scale included problems such as the presence of litter, graffiti, public drinking, and abandoned buildings. All items comprising this sub-scale were responded to using a yes/no format yielding a possible range of 0 to 7. The internal consistency reliability of this sub-scale was 0.66. Total scores on this subscale were calculated by summing scores for all of the items.

The NCQ has been used in a wide variety of studies to collect community members' perceptions of the safety and quality of their local neighbourhoods. Past research suggests that the measure has adequate reliability and validity [41].

#### Demographic data

A variety of information on students' demographic features, including their age and gender, was collected using a series of brief questions. Students were asked to report their exact age in years on their last birthday and their gender. Students were also asked to report on their mothers' highest level of education using the categories of no schooling, kindergarten, primary, secondary/high school, trade/vocational, associate degree, bachelor's degree and graduate degree. For the data analyses, these categories were collapsed into secondary education or below or post-secondary education. Maternal education was used as an indicator of social class in some of our analyses.

### Procedure

#### Ethical and institutional approvals

We obtained ethical approval for this research project from the Faculty of Medical Sciences Ethics Committee of The University of the West Indies – Mona. The research proposal noted that the research was to be conducted in four separate Caribbean countries. Permission to conduct the study was obtained from the Ministry of Education in each country. Further, the approval of the principal of each school where data were collected was obtained prior to the data collection. Written informed consent was obtained from the parents or guardians of each student in the research study via the informed consent form approved by the Faculty of Medical Sciences Ethics Committee of The University of the West Indies – Mona. In addition, informed assent to take part in the study was obtained from each student prior to data collection using the informed assent form approved by the Faculty of Medical Sciences Ethics Committee of The University of the West Indies – Mona.

#### Data collection

Prior to the start of the project, a research assistant liaised with the Ministry of Education in each country and the schools selected to participate in the project in order to describe the study's aims and obtain consent for conducting the project. Students in the classrooms selected for this project were given an informed consent form for their parents to complete. Data for the project were collected from students during one of their regularly scheduled classes. All students in a classroom whose parents provided their informed consent for their adolescent to take part in the project were given a package of instruments to complete. This package consisted of an informed consent form for the adolescents, the BDI-II, the Neighbourhood Characteristics Questionnaire and a series of questions regarding their demographic features.

### Statistical Analyses

A variety of simple and more complex analyses were used in the study. First, to determine the representativeness of our sample, the gender distribution of our sample was compared to Official statistics from UNESCO [23] for the same grade level and year. Next the prevalence of BDI-II depression scores by country was estimated using frequency analyses. To explore the base relationship of neighbourhood factors to depressive symptoms, the correlation of each neighbourhood factor to BDI-II depression scores was calculated. To examine differences between the countries by neighbourhood factors we conducted a series of one-way ANOVA analyses – one for each neighbourhood factor. To test our hypotheses, we conducted a series of hierarchical multiple regression analyses. Prior to conducting these regression analyses, we checked for evidence of singularity and multicollinearity of the neighbourhood factors by calculating the inter-correlation of the five neighbourhood factors and by conducting a series of multiple regression analyses whereby each neighbourhood factor was predicted by the other four neighbourhood factors. Following the exploration of multicollinearity, a series of two stage hierarchical multiple regression analyses were conducted using the neighbourhood factors as predictors of BDI-II depression scores. Separate regression analyses were conducted for each of the four islands. In these analyses, background factors (gender, age, and maternal education) were first entered into the regression equation, followed by each of the five neighbourhood factors. To explore the interactive effects of island by neighbourhood factors, we conducted another series of hierarchical multiple regression analyses. In these analyses the background variables of gender, age and maternal education were first entered into the regression equation, followed at the second stage by the dummy coded variables representing island, with Jamaica used as the reference group, and a single neighbourhood factor. At the third stage of the regression analyses the country by neighbourhood factor interactions were entered into the equation. Gender was statistically controlled for as previous research has indicated that gender is a significant predictor of depression scores. Age was used as a statistical control as it is known that depression differs by age. Education was used as a proxy for socio-economic status as educational levels is strongly associated with occupational attainment and family income[42]. Maternal education was used as a proxy for social class as nearly half of all households in the islands we included in our study are headed by single women [43]–[45].

## Results

### Comparability of the Obtained Sample

To determine the representativeness of our sample, the gender distribution of the obtained sample was compared to data from UNESCO for the same grade level and year [23]. The UNESCO dataset was used to determine representativeness as it contained data for all four islands for the year 2007. Information was only available for students' gender within the UNESCO data, making only gender as a point of comparison. Examining the distributions (Table 2), it can be seen that the obtained sample of students approximated that of the official reported gender distribution for each Island. Overall, with the exception of St. Vincent where it closely matched, the gender distribution of the obtained sample for each island had between 2.6% to 3.8% more females than the officially reported population.

**Table 2:** Comparison of the Official School Population by Island to the Obtained School Population by Country.

		UNESCO		Current Sample
Country	Total Enrolment	Total Females	Percent Females	Percent Females
Jamaica	7,556	3,659	48.4	52.2
Bahamas	5,600	2,795	49.9	52.5
St. Kitts and Nevis	913	427	46.8	50.3
St. Vincent	1,959	1,049	53.5	53.6

### Prevalence of Depression

The prevalence of depression was examined using simple frequency and cross-tabulations (Table 3). Across the four islands, students in Jamaica reported the highest levels of depressive symptoms while students in the Bahamas and St. Kitts and Nevis reported the lowest levels (χ^2^ (9) = 48.17, p<0.05). Nearly two-thirds (64%) of all students in Jamaica reported some level of depressive symptoms with 23.4% reporting mild levels, 26.3% reporting moderate levels, and 14.4% reporting severe levels of depressive symptoms. Just over half (55.4%) of the students in St. Vincent reported some level of depressive symptoms with 24.4% reporting mild, 21.8% reporting moderate, and 9.2% reporting severe levels of depressive symptoms. In contrast, less than half of the students in the Bahamas (45.6%) reported some symptoms of depression, with 23% reporting mild, 15.7% reporting moderate, and 6.9% reporting severe symptoms of depression. Similarly, only 46.3% of students in St. Kitts and Nevis reported some symptoms of depression, with 21.6% reporting mild, 14.0% reporting moderate, and 10.7% reporting severe symptoms of depression.

**Table 3:** Depressive symptoms by country.

Country						
		Overall Depressive Symptoms	Minimal	Mild	Moderate	Severe
Jamaica	%	64.0%	36.0%	23.4%	26.3%	14.4%
	n	178	100	65	73	40
Bahamas	%	45.6%	54.4%	23.0%	15.7%	6.9%
	n	99	118	50	34	15
St. Kitts and Nevis	%	46.3%	53.7%	21.6%	14.0%	10.7%
	n	341	396	159	103	79
St. Vincent	%	55.4%	44.6%	24.4%	21.8%	9.2%
	n	397	319	175	156	66

Chi-Square Test = 48.17, p<.001

### Correlation of Neighbourhood Factors to Depressive Symptoms

Overall, neighbourhood factors had the strongest association with depressive symptoms for students from Jamaica, followed by those from St. Kitts and Nevis (Table 4). Neighbourhood attachment was negatively correlated to depressive symptoms for all countries, having a moderate relationship for all, except the Bahamas. The more attached students were to their neighbourhoods, the lower their reported depressive symptoms. Poor neighbourhood quality was positively correlated to depressive symptoms such that the poorer the perceived quality of the neighbourhood, the higher the depressive symptoms students reported. This correlation was small for students from all islands except Jamaica where it had a moderate association. Perceived levels of neighbourhood crime had a small relationship to students' reported depressive symptoms for all islands. Perceived neighbourhood disorder had a differential relationship with students' reported depressive symptoms across each of the four islands with this association being moderate for Jamaica, small for St. Vincent and St. Kitts and Nevis and near zero for the Bahamas. Students' perceptions of social cohesion were negatively associated with their reported depressive symptoms such that the less socially cohesive they perceived their neighbourhoods, the higher were their reported depressive symptoms. This correlation was largest for Jamaica, smaller for the Bahamas and St. Kitts and Nevis, and near zero for St. Vincent.

**Table 4:** Correlation of Neighbourhood Factors to Depressive Symptoms.

BDI - II Scores				
Neighbourhood Factor	Jamaica	The Bahamas	St. Kitts	St. Vincent
Poor Quality	.31*	.16*	.15*	.16*
Crime	.20*	.21*	.17*	.13*
Disorder	.31*	.03	.20*	.14*
Attachment	−.32*	−.15*	−.27*	−.26*
Social Cohesion	−.24*	−.13*	−.10*	−.06

Note: n = 1948

All tests of significance were conducted using t-tests of the correlation coefficents

* p<.01

### Perceptions of Neighbourhood Characteristics

A series of one-way ANOVA's were conducted to explore differences by country in adolescents' perceptions of the characteristics of their neighbourhoods. Consistent with expectations, significant differences were found in adolescents' perceptions of the social cohesiveness of their neighbourhoods (F(3, 1944) = 17.32, p<.01). Bonferroni post-hoc tests indicated that Jamaican adolescents perceived their neighbourhoods to be significantly more socially cohesive than adolescents in the Bahamas, St. Kitts and Nevis, and St. Vincent. Significant differences across the countries were also found for adolescents' perceived quality of their neighbourhoods (F(3, 1944) = 20.96, p<.01). Bonferroni post-hoc tests also indicated that adolescents in St. Vincent perceived their neighbourhoods to be significantly poorer in quality than those in Jamaica, the Bahamas, and St. Kitts and Nevis (Table 5). Adolescents differed in their perceptions of the amount of crime in their neighbourhoods across countries (F(4,1944) = 4.69, p<.01). Bonferroni post-hoc tests indicated that Jamaican adolescents perceived their neighbourhoods to have lower levels of crime than adolescents in St. Vincent or St. Kitts and Nevis (Table 5). Perceptions of neighbourhood disorder differed across the countries (F(3, 1944) = 14.29, p<.01). Jamaican adolescents perceived their neighbourhoods to be significantly less disordered than adolescents in the Bahamas, St. Vincent, and St. Kitts and Nevis (Table 5). Finally, adolescents differed in their attachment to their neighbourhoods (F(3, 1944) = 14.00, p<.01). Post-hoc tests indicated that adolescents in Jamaica and the Bahamas were significantly more attached to their neighbourhoods than adolescents in St. Vincent or St. Kitts and Nevis (Table 5).

**Table 5:** Mean perceived neighbourhood factors by country†.

		Neighbourhood Social Cohesion	Poor Neighbourhood Quality	Neighbourhood Crime	Neighbourhood Disorder	Neighbourhood Attachment‡
		Mean (sd)	Mean (sd)	Mean (sd)	Mean (sd)	Mean (sd)
Country	Jamaica	4.99 (1.78)	8.96 (2.95)	2.89 (2.42)	2.60 (1.81)	19.04 (4.28)
	Bahamas	4.32 (1.93)^a^	9.03 (2.60)	3.35 (2.44)	3.17 (1.92)^a^	18.42 (4.68)
	St. Kitts and Nevis	4.48 (1.81)^a^	8.81 (2.77)	3.39 (2.45)^a^	3.34 (1.97)^a^	20.25 (4.20)^ab^
	St. Vincent	4.09 (1.83)^ac^	9.97 (3.17)^abc^	3.52 (2.35)^a^	3.41 (1.66)^a^	20.10 (4.32)^ab^
	F-Test	17.32**	20.96**	4.69*	14.29**	14.00**

† Tests of Mean Differences were conducted using Bonferroni post-hoc tests ANOVA.

‡ Low scores on Neighbourhood Attachment indicate higher levels of attachment.

a Different from Jamaica

b Different from Bahamas

c Different from St. Kitts and Nevis

d Different from St. Vincent

sd =  Standard Deviation

* p<.005

** p<.001

### Regression analyses of island and neighbourhood factors predicting depressive symptoms

As mentioned previously, a series of two stage hierarchical multiple regression analyses were conducted using the neighbourhood factors as predictors of BDI-II depression scores. Separate regression analyses were conducted for each of the four islands. In these analyses, background factors (gender, age, and maternal education) were first entered into the regression equation, followed by each of the five neighbourhood factors. Before conducting these analyses an examination was made for singularity and multicollinearity among the neighbourhood factors by calculating the simple correlations of the neighbourhood factors to each other and by performing a series of multiple regression analyses whereby each neighbourhood factor was predicted by all of the other neighbourhood factors. All of the neighbourhood factors had correlations with each other which were lower than 0.50. As such, none of the neighbourhood factors were near perfectly correlated with each other, indicating an absence of singularity (Table 6). Results of the regression analyses (Table 7) suggest that the neighbourhood factors were not multicolinear.

**Table 6:** Inter-correlation of Neighbourhood Factors.

	Attachment	Social Cohesion	Quality	Crime	Disorder
Attachment	1.0				
Social Cohesion	.33*	1.0			
Quality	−.35*	−.24*	1.0		
Crime	−.21*	−.10*	.47*	1.0	
Disorder	−.16*	−.08*	.44*	.49*	1.0

Note: n = 1948

All tests of significance were conducted using t-tests of the correlation coefficents

* p<.01

**Table 7:** Squared Multiple Correlation Coefficients of Each Neighbourhood Sub-Scale predicted by the Other Neighbourhood Sub-Scales.

Neighbourhood Sub-Scale	R^2^
Quality	.35
Crime	.32
Disorder	.30
Attachment	.19
Social Cohesion	.13

Controlling for age, gender and social class, neighbourhood factors as a group were significant predictors for all islands (Tables 8, 9, 10, 11). However, these relationships were not equally strong for all of the islands with perceived neighbourhood factors accounting for the greatest amount of variance in students' reported depressive symptoms for Jamaica (R^2^ = .181) followed by St. Kitts and Nevis (R^2^ = .078), St. Vincent (R^2^ = .070), and the Bahamas (R^2^ = .063).

**Table 8:** Multiple regression analyses of neighbourhood factors predicting BDI-II depression scores, controlling for gender, maternal education and students' age, for Jamaica.

	Predictor	B	Beta	t-test	Chg R^2^
Stage One	Control				
	Gender	.244	.01	.18	0.005
	Maternal Education	−1.05	−.05	−.78	
	Age	.77	.04	.68	
Stage Two	Main Effects				
	Neighbourhood Attachment	−.47	−.19	−2.77*	0.181*
	Neighbourhood Social Cohesion	−.75	−.13	−2.06*	
	Neighbourhood Quality	.36	.10	1.34	
	Neighbourhood Crime	.06	.01	.19	
	Neighbourhood Disorder	1.18	.20	2.79*	

Note: n = 1948

All tests of significance for the Change in R^2^ were conducted using F-tests

* p≤0.05

**Table 9:** Multiple regression analyses of neighbourhood factors predicting BDI-II depression scores, controlling for gender, maternal education and students' age, for the Bahamas.

	Predictor	B	Beta	t-test	Chg R^2^
Stage One	Control				
	Gender	4.48	.24	3.47	0.079*
	Maternal Education	−1.43	−.07	−1.09	
	Age	1.62	.15	2.52*	
Stage Two	Main Effects				
	Neighbourhood Attachment	−.03	−.02	−.19	0.063*
	Neighbourhood Social Cohesion	−.31	−.06	−.86	
	Neighbourhood Quality	.44	.12	1.53	
	Neighbourhood Crime	.75	.20	2.27*	
	Neighbourhood Disorder	−.61	−.13	−1.54	

Note: n = 1948

All tests of significance for the Change in R^2^ were conducted using F-tests

* p≤0.05

**Table 10:** Multiple regression analyses of neighbourhood factors predicting BDI-II depression scores, controlling for gender, maternal education and students' age, for St. Kitts and Nevis.

	Predictor	B	Beta	t-test	Chg R^2^
Stage One	Control				
	Gender	5.21	.25	6.61	0.078*
	Maternal Education	−2.02	−.09	−2.37*	
	Age	.57	.04	1.16*	
Stage Two	Main Effects				
	Neighbourhood Attachment	−.53	−.22	−5.16*	0.078*
	Neighbourhood Social Cohesion	−.03	−.01	−.86	
	Neighbourhood Quality	−.03	−.01	−.17	
	Neighbourhood Crime	.24	.06	1.27	
	Neighbourhood Disorder	.52	.09	2.19*	

Note: n = 1948

All tests of significance for the Change in R^2^ were conducted using F-tests

* p≤0.05

**Table 11:** Multiple regression analyses of neighbourhood factors predicting BDI-II depression scores, controlling for gender, maternal education and students' age, for St. Vincent.

	Predictor	B	Beta	t-test	Chg R^2^
Stage One	Control				
	Gender	5.19	.28	7.29	.097*
	Maternal Education	−1.73	−.08	−2.14*	
	Age	1.12	.12	3.03*	
Stage Two	Main Effects				
	Neighbourhood Attachment	−.51	−.23	−5.79*	0.070*
	Neighbourhood Social Cohesion	.34	.07	1.72	
	Neighbourhood Quality	.21	.07	1.55	
	Neighbourhood Crime	.05	.01	.31	
	Neighbourhood Disorder	.20	.04	.85	

Note: n = 1948

All tests of significance for the Change in R^2^ were conducted using F-tests

* p≤0.05

For the Jamaican sample perceived neighbourhood attachment, social cohesion and disorder were significant predictors of students' reported depressive symptoms (Table 8). With regard to the Bahamian cohort only perceived neighbourhood crime was a significant predictor of students' reported depressive symptoms (Table 9). Regarding students from St. Kitts and Nevis, perceived neighbourhood attachment and disorder were significant predictors of students' reported depressive symptoms (Table 10) and for those from St. Vincent only neighbourhood attachment was a significant predictor (Table 11).

### The interactive relationships of island and neighbourhood factors with depressive symptoms

Exploring the interactive effects of island by neighbourhood factors, we conducted another series of hierarchical multiple regression analyses. In these analyses the background variables of gender, age and maternal education were first entered into the regression equation, followed at the second stage by the dummy coded variables representing island, with Jamaica used as the reference group, and a single neighbourhood factor. At the third stage of the regression analyses the country by neighbourhood factor interactions were entered into the equation.

Using Jamaica as the reference group, only some of the interactions of the islands by neighbourhood factors were significant predictors of students' reported depressive symptoms (Tables 12, 13, 14, 15, 16). The interactions of islands by perceived neighbourhood disorder, attachment, and social cohesion were statistically significant. With regard to perceived neighbourhood disorder, the interactions of the Bahamas and St. Vincent were statistically significant (Table 14) such that for the same level of perceived neighbourhood disorder, adolescents from both of these islands reported significantly lower depressive symptoms than students from Jamaica. Similarly, considering perceived neighbourhood attachment, only the interaction for the Bahamian students was statistically significant (Table 15), such that for the same level of perceived neighbourhood attachment, Bahamian students reported significantly higher levels of depressive symptoms than their Jamaican peers. Finally, perceived social cohesion had a statistically significant relationship for teenagers from St. Vincent and St. Kitts and Nevis (Table 16), such that for the same level of perceived neighbourhood cohesion, adolescents from these countries reported higher levels of depressive symptoms than those from Jamaica.

**Table 12:** Multiple regression analyses of country and neighbourhood quality predicting BDI-II depression scores, controlling for gender, maternal education and students' age.

	Predictor	B	Beta	t-test	Chg R^2^
Stage One	Control				
	Gender	4.47	.22	9.62*	0.063*
	Maternal Education	−1.37	−.07	−2.79*	
	Age	.83	.08	3.31*	
Stage Two	Main Effects				
	St. Kitts	−3.22	−.16	−4.50*	0.042*
	Bahamas	−2.60	−.08	−2.81*	
	St. Vincent	−2.89	−.14	3.98*	
	Neighbourhood Quality	.60	.18	7.71*	
Stage Three	Interactions				
	St. Kitts by Poor Neighbourhood Quality	−.55	−.25	−2.25*	0.004
	Bahamas by Poor Neighbourhood Quality	−.43	−.13	−1.30	
	St. Vincent by Poor Neighbourhood Quality	−.60	−.31	−2.57*	

Note: n = 1948

All tests of significance for the Change in R^2^ were conducted using F-tests

* p≤0.05

**Table 13:** Multiple regression analyses of country and neighbourhood crime predicting BDI-II depression scores, controlling for gender, maternal education and students' age.

	Predictor	B	Beta	t-test	Chg R^2^
Stage One	Control				
	Gender	4.47	.22	9.62*	0.063*
	Maternal Education	−1.37	−.07	−2.79*	
	Age	83	.08	3.31*	
Stage Two	Main Effects				
	St. Kitts	−3.62	−.18	−5.02*	0.033*
	Bahamas	−2.87	−.09	−3.08*	
	St. Vincent	−2.68	−.13	3.67*	
	Neighbourhood Crime	.61	.15	6.46*	
Stage Three	Interactions				
	St. Kitts by Neighbourhood Crime	−.08	−.02	−.27	0.001
	Bahamas by Neighbourhood Crime	−.04	−.01	−.10	
	St. Vincent by Neighbourhood Crime	−.35	−.08	−1.18	

Note: n = 1948

All tests of significance for the Change in R^2^ were conducted using F-tests

* p≤0.05

**Table 14:** Multiple regression analyses of country and neighbourhood disorder predicting BDI-II depression scores, controlling for gender, maternal education and students' age.

	Predictor	B	Beta	t-test	Chg R^2^
Stage One	Control				
	Gender	4.47	.22	9.62*	0.063*
	Maternal Education	−1.37	−.07	−2.79*	
	Age	83	.08	3.31*	
Stage Two	Main Effects				
	St. Kitts	−3.90	−.19	−5.40*	0.035*
	Bahamas	−3.06	−.10	−3.28*	
	St. Vincent	−2.93	−.14	−4.01*	
	Neighbourhood Disorder	.84	.16	6.68*	
Stage Three	Interactions				
	St. Kitts by Neighbourhood Disorder	−.69	−.14	−1.80	0.005*
	Bahamas by Neighbourhood Disorder	−1.46	−.17	−3.01*	
	St. Vincent by Neighbourhood Disorder	−.97	−.19	−2.43*	

Note: n = 1948

All tests of significance for the Change in R^2^ were conducted using F-tests

* p≤0.05

**Table 15:** Multiple regression analyses of country and neighbourhood attachment predicting BDI-II depression scores, controlling for gender, maternal education and students' age.

	Predictor	B	Beta	t-test	Chg R^2^
Stage One	Control				
	Gender	4.47	.22	9.62*	0.063*
	Maternal Education	−1.37	−.07	−2.79*	
	Age	83	.08	3.31*	
Stage Two	Main Effects				
	St. Kitts	−2.79	−.14	−3.95*	0.070*
	Bahamas	−2.80	−.09	−3.07*	
	St. Vincent	−1.87	−.09	−2.62*	
	Neighbourhood Attachment	−.56	.25	−10.90*	
Stage Three	Interactions				
	St. Kitts by Neighbourhood Attachment	.20	.20	1.23	0.005*
	Bahamas by Neighbourhood Attachment	.61	.37	3.10*	
	St. Vincent by Neighbourhood Attachment	29	.29	1.79	

Note: n = 1948.

All tests of significance for the Change in R^2^ were conducted using F-tests.

* p≤0.05.

**Table 16:** Multiple regression analyses of country and social cohesion predicting BDI-II depression scores, controlling for gender, maternal education and students' age.

	Predictor	B	Beta	t-test	Chg R^2^
Stage One	Control				
	Gender	4.47	.22	9.62*	0.063*
	Maternal Education	−1.37	−.07	−2.79*	
	Age	.83	.08	3.31*	
Stage Two	Main Effects				
	St. Kitts	3.65	−.18	−5.02*	0.020*
	Bahamas	−2.83	−.09	−3.01*	
	St. Vincent	−2.84	−.14	−3.83*	
	Neighbourhood Social Cohesion	−.50	.09	−3.94*	
Stage Three	Interactions				
	St. Kitts by Neighbourhood Social Cohesion	1.01	.25	2.55*	0.005*
	Bahamas by Neighbourhood Social Cohesion	.94	.14	1.92	
	St. Vincent by Neighbourhood Social Cohesion	1.26	.29	3.18*	

Note: n = 1948.

All tests of significance for the Change in R^2^ were conducted using F-tests.

* p≤0.05.

## Discussion

Adolescents' perceptions of their neighbourhoods appear to be differentially associated with their emotional health across the four countries. The relationship between island of residence and depressive symptoms may be moderated by students' perceptions of the their neighbourhoods, such that for the Jamaican cohort, positive perceptions of the students' neighbourhoods played a greater role in attenuating their depressive symptoms than students in the St. Vincent and St. Kitts and Nevis. Specifically, higher levels of social cohesion were associated with greater reductions in depressive symptoms among Jamaican adolescents than their peers in the other islands, while for similar levels of attachment to their neighbourhoods, Bahamian students reported higher levels of depressive symptoms than Jamaicans. However, greater perceptions of neighbourhood disorder were associated with higher levels of depressive symptoms among Jamaican adolescents than their peers in the Bahamas and St Vincent. In general, the social context of neighbourhoods appears to be important for Caribbean adolescents.

### The social context and subjective perceptions of neighbourhood factors reduce Jamaican adolescents' symptoms of depression

Our research found that the relationship between island of residence and depressive symptoms was moderated by students' perceptions of their neighbourhoods, such that for Jamaica, positive perceptions of the students' neighbourhoods played a greater role in attenuating their depressive symptoms than students in St. Vincent and St. Kitts and Nevis. One possible explanation for this finding is that the social structure of the neighbourhoods differs across Caribbean Islands. The differences in the patterns of living arrangements and family structures lead to different perceptions of neighbourhood. In situations where community members must rely on each other for their joint survival there are higher levels of cooperation and increased social bonds. One factor that distinguishes Jamaica from the other Caribbean islands is the presence of tenement yards [46], [47]. These yards are large lots on which multiple families live and share resources, including food and other amenities – as well as cooperate to solve problems and to socialize [47]. In contrast, in St. Vincent and St. Kitts and Nevis there are very few tenement yards, if any, and the persons who live in these yards tend to be family members [47]. This set of conditions in Jamaica creates a situation where the communal nature of neighbourhoods provides adolescents with some social support even if their neighbourhood conditions are poor, thus producing more positive perceptions of their communities.

In contrast, perceptions of neighbourhood disorder were more strongly associated with depressive symptoms among Jamaican adolescents. This suggests that positive perceptions of neighbourhood order play an important protective role in the emotional health of adolescents, especially for those from Jamaica.

Jamaican adolescents, across the aspects of the neighbourhoods that we assessed, perceived their neighbourhoods more positively than adolescents in the other countries. Moreover, the correlation of neighbourhood factors to depressive symptoms was strongest for them. It seems that adolescents from the Bahamas, St. Kitts and Nevis, and St. Vincent may be using other strategies to protect them from developing intense symptoms of depression.

### The social context of neighbourhoods and depressive symptoms among adolescents from St. Vincent and St. Kitts and Nevis

The differences in attachment to communities may be explained by migration practices and intentions. This study found that Bahamian and Jamaican adolescents were more attached to their neighbourhoods than students from St. Vincent and St. Kitts and Nevis. Many residents of St. Vincent who migrate, tend not to return home [48]. This may be due to fewer chances for upward mobility or employment in St Vincent [48]. Coupled with the limited social assistance programmes, this may be associated with less attachment to the lifestyle in St. Vincent [49] and social cohesion in the country.

In St. Kitts and Nevis there is an increasing trend for residents to marry people living abroad and to share their time between two different countries, [14], [50]. Dividing families attention between two different countries may serve to decrease opportunities for social networking within their communities, ultimately leading to reduced attachment to neighbourhoods [14], [50]. In addition, there is an increasing tendency to move away from extended families settings and towards nuclear or single parent homes [14], [50]. These factors have led to a decline in the ethos of having a village raise a child [14], [50]. Consequently, there is less emphasis on communal involvement and social cohesion leading to decreased efforts towards activities such as neighbours doing favours for each other, watching each other's properties, or joining together to solve community problems.

In general, the association between perceptions of neighbourhood factors and depressive symptoms was smallest amongst the Bahamian students. One possible explanation for this is the Basking in Reflected Glory effect [51]. This effect occurs when individuals experience increased self-esteem through their association with successful people. Bahamians identify strongly with the culture and society of the United States. The United States is generally seen by them as a powerful and successful nation and as such, it is possible that Bahamian students may have elevated levels of high self-esteem which serves to attenuate the relationship of their perceived neighbourhood factors to depressive symptoms.

### Perceptions versus Reality in Neighbourhood Factors

Past research [52] has suggested that perceptions of neighbourhood factors play a stronger role in predicting young adults' mental health than the physical conditions of communities. In the current study we asked adolescents to provide us with their perceptions of the quality of their neighbourhoods. Despite the high levels of crime, and unemployment [53]–[55] in Jamaican society, Jamaican adolescents had the most favourable perceptions of their neighbourhoods. This may explain why neighbourhood factors had a stronger association with lower levels of depressive symptoms for Jamaican adolescents than for adolescents in the other islands.

### Future research

One implication of this research is that Jamaican adolescents may not experience depressive symptoms in the same way as adolescents in the other islands, as they preserve their perceptions of their neighbourhood and society. Future research should examine differential manifestations of depressive symptoms among adolescents across Caribbean islands.

In the future, social policy makers may wish to focus on improving students' perceptions of their neighbourhoods, communities and national identity as this may help to reduce the negative impact of social conditions on adolescents' emotional health. Traditionally governments have attempted to change the objective social conditions. The authors of this paper would like to suggest that an additional goal should be to improve adolescents' perceptions of their environment.

### Limitations

This project had several key limitations. Data on neighbourhood factors and depressive symptoms were collected at only one point in time, limiting our ability to establish a causal association of neighbourhood factors with depressive symptoms. Further, students completed a self-report measure of depressive symptoms. Therefore, it was not possible to determine if students were clinically depressed. Because the data for this project were collected from students on only one school day, children who wished to avoid participation in the research may not have attended their schools on the day of data collection. Additionally, students were not sampled from all islands comprising the Bahamas or St. Vincent and the Grenadines. Only students from New Providence Island in the Bahamas and St. Vincent were included in this research project. Consequently, the findings reported here may not be representative of the other islands of the Bahamas or the Grenadines. Finally, the study sampled only adolescents attending school. Adolescents attending schools tend to come from more structured communities, with families who have higher expectations of them. As such, these adolescents may not be completely representative of all adolescents in each of the countries.

## Conclusion

Neighbourhood factors appear to be moderate adolescents' self-reports of depressive symptoms. However, other factors may mitigate this relationship.
